# Rethinking the pros and cons of randomized controlled trials and observational studies in the era of big data and advanced methods: a panel discussion

**DOI:** 10.1186/s12919-023-00285-8

**Published:** 2024-01-18

**Authors:** Pamela Fernainy, Alan A. Cohen, Eleanor Murray, Elena Losina, Francois Lamontagne, Nadia Sourial

**Affiliations:** 1 Department of Health Management, Evaluation and Policy, School of Public Health, University of Montreal, Montreal, QC Canada; 2 Research Centre of the Centre Hospitalier de L’Université de Montréal (CHUM), Montreal, QC Canada; 3 Department of Family and Emergency Medicine, Faculty of Medicine and Health Sciences, University of Sherbrooke, Montreal, QC Canada; 4 CHUS Research Centre, Montreal, QC Canada; 5 Centre de Recherche Sur Le Vieillissement, Montreal, QC Canada; 6 Butler Columbia Aging Center, New York, NY USA; 7 Department of Environmental Health Sciences, Mailman School of Public Health, Columbia University New York, New York, USA; 8 School of Public Health, Boston University, Boston, MA USA; 9 Harvard Medical School Department of Orthopedic Surgery, Cambridge, MA USA; 10 Departement de Medicine, University of Sherbrooke, Montreal, QC Canada

**Keywords:** Randomized controlled trial, Randomized control trial, Observational study, Medical evidence, Research method, Research methodologies, Big data, Innovation, Study design, Quality of evidence

## Abstract

Randomized controlled trials (RCTs) have traditionally been considered the gold standard for medical evidence. However, in light of emerging methodologies in data science, many experts question the role of RCTs. Within this context, experts in the USA and Canada came together to debate whether the primacy of RCTs as the gold standard for medical evidence, still holds in light of recent methodological advances in data science and in the era of big data. The purpose of this *manuscript,* aims to raise awareness of the pros and cons of RCTs and observational studies in order to help guide clinicians, researchers, students, and decision-makers in making informed decisions on the quality of medical evidence to support their work. In particular, new and underappreciated advantages and disadvantages of both designs are contrasted. Innovations taking place in both of these research methodologies, which can blur the lines between the two, are also discussed. Finally, practical guidance for clinicians and future directions in assessing the quality of evidence is offered.

**Supplementary Information:**

The online version contains supplementary material available at 10.1186/s12919-023-00285-8.

## Background

Randomized controlled trials (RCTs) have traditionally been considered the gold standard for medical evidence because of their ability to eliminate bias due to confounding and to thereby ensure internal validity [1]. However, the primacy of RCTs is far from universally accepted by methodological experts. This is particularly true in the era of big data and in light of emerging methodologies in data science, machine learning, causal inference methods, and other research methods, which may shift how researchers view the relative quality of evidence from observational studies compared to RCTs. In this context, on February 24, 2022, a debate took place to discuss the pros and cons of randomized control trials and observational studies. This debate was intended to reach a wide audience at all levels of training and expertise, and welcomed clinicians, researchers, students, and decision-makers seeking to better navigate the complex landscape of health evidence in a fast-changing world. The webinar announcement was shared through multiple research centers and the social networks of the panelists. A broad range of attendees participated (total of 267 attendees: 35% researchers, 28% students, 16% clinicians, 5% managers and 15% other), with varying levels of methodological expertise (26% minimal, 56% moderate, and 18% advanced). The panel was composed of clinicians and researchers with methodological expertise in experimental and observational studies from the USA and Canada (authors AAC, EM, EL, FL, and NS). This article seeks to summarize areas of agreement and disagreement among discussion panelists, highlight methodological innovations, and guide researchers, students, decision-makers, and clinicians in making informed decisions on the quality of medical evidence. The debate can be viewed at https://www.youtube.com/watch?v=VNc30fab9nM&t=17s. A lay infographic of the key points of the debate is also available (Appendix A).

## Main body

In general, RCTs are studies where investigators randomly assign subjects to different treatment groups (intervention or control group) to examine the effect of an intervention on relevant outcomes [2]. In large samples, random assignment generally results in balance between both observed (measured) and unobserved (unmeasured) group characteristics [1]. In observational studies, investigators observe the effects of exposures on outcomes using either existing data such as electronic health records (EHRs) [3], health administrative data, or collected data such as through population-based surveys [4]. Thus, in observational studies, the investigator does not play a role in the assignment of an exposure to the study subjects [5].

### Pros and cons of RCTs and observational studies

By and large, RCTs are well suited to establish the efficacy of interventions involving medical interventions, and can accordingly advance knowledge that is important to the work of clinicians and the subsequent improvement of patients’ well-being. Besides being prescriptive and intuitive, the key feature of RCTs is the control for confounding due to the random assignment of the exposure of interest. Under ideal conditions, this design ensures high internal validity and can provide an unbiased causal effect of the exposure on the outcome [6]. Consequently, RCTs are helpful to physicians who prescribe medications, and studies that deal with medications as interventions lend themselves to such studies. Conversely, the lack of random assignment in observational studies is a key disadvantage, opening up the possibility of bias due to confounding and requiring researchers to employ more sophisticated methods when attempting to control for this important source of bias [7]. For instance, when considering the effect of alcohol consumption on lung cancer, factors such as smoking should be considered, as smoking has been linked to both alcohol consumption and lung cancer and can therefore confound the effect of interest if not controlled. Yet, in reality, generalizability of RCTs may also be threatened due to selection bias [8] or particularities of the study population. Furthermore, randomization of the exposure only protects against confounding at baseline [9]. Confounding might occur during the course of the study, due to loss to follow up, non-compliance, and missing data [10, 11]. These post-randomization biases are often overlooked and the benefits of randomization at baseline may give researchers and clinicians a false sense of security.

Conversely, in observational studies, researchers are keenly aware of the threat to validity due to bias and must often consider and implement methods at the design, analysis and interpretation stage to account for it [12]. An advantage of observational studies is that they allow researchers to examine the effect of natural experiments including the effect of interventions under real-world conditions [13, 14]. This is particularly relevant when the study system is formally complex, such as for physiological and biochemical regulatory networks, healthcare systems, infectious diseases, and social networks. In this case, results may be highly contingent on many factors, for example, when assessing COVID-19 public health measures during the pandemic, determining the impact of lifestyle, or a patient belonging to an interprofessional primary care team. In these contexts, observational studies may provide better external validity than RCTs, which typically occur under well-controlled and, by the same token, often less realistic conditions. Observational studies are also preferred when RCTs are too costly, not feasible, time-intensive, or unethical to conduct [13]. For example, a RCT studying the development of melanoma would require a long follow-up period and may not be feasible. Among researchers, there is overall agreement that low-quality RCTs might not be generally superior to observational studies, but disagreement remains as to whether high-quality RCTs, as a rule, provide a higher standard of evidence [13]. For panelists, this disagreement stemmed partly from the relative weights they accorded to internal versus external validity. While no panelist felt that observational studies were systematically better than RCTs, there was disagreement as to whether the notion that RCTs are a gold standard is helpful or harmful. Still, despite this disaccord, methodological advances are opening the door to promising opportunities. Table 1 provides a succinct summary of several pros and cons of RCTs and observational studies.

**Table 1:** Pros and cons of randomized control trials and observational studies

	Randomized control trials	Observational studies
Pros	• Random assignment makes study groups similar and comparable (no confounding at baseline) • Best fit to establish the efficacy of pharmacologic interventions • Currently considered as the gold standard for studying the effect of an intervention • Based on clear and well-established guidelines • Gives the true effect of an intervention under ideal conditions (internal validity)	• Useful to provide real-world evidence (external validity) • Relatively fast and inexpensive to conduct when data is already available • May take advantage of already available data like electronic health records • Suitable for studies where randomization is not ethical, or not feasible (e.g. rare diseases)
Cons	• Can be costly and take many years to conduct • Data collected may be biased due to non-compliance and drop-outs (post-randomization bias) • Possible to overlook biases • Generalizable only in simple systems, or when the conditions are exactly replicated	• Subject to outside factors that could distort the effect of the intervention (confounding) • Can be complex to design • Advanced analytical approaches are often required • Subject to limitations in the data available

### Innovations and opportunities in RCTs and observational studies

Recent innovations in RCTs have facilitated or improved the results of this research method and can result in trials that are more flexible, efficient, or ethical [15]. New designs being considered in RCTs include, but are not limited to, adaptive trials, sequential trials, and platform trials. Adaptive trials, for instance, include scheduled interim looks at the data during the trial. This leads to predetermined changes based on the analyses of accumulating data, all the while maintaining trial validity and integrity [15]. Sequential trials are an approach to clinical trials during which subjects are serially recruited and study results are continuously analyzed [16]. Once enough data enabling a decision regarding treatment effectiveness is collected, the trial is stopped [17]. Platform trials focus on an entire disease or syndrome to compare multiple interventions and add or drop interventions over time [18]. Also, the development of EHRs and an expanded access to routinely-collected clinical data has resulted in RCTs being conducted within the context of EHR-based clinical trials. EHRs have the potential to advance clinical health research by facilitating RCTs in real-world settings. Many RCTs have leveraged EHRs to recruit patients or assess clinical outcomes with minimal patient contact [19]. Such approaches are considered a particularly innovative convergence of observational and experimental data, which blurs the line between these two methodologies going forward.

As well as innovations in RCTs, innovations are taking place in observational studies. The last two decades have seen the use of novel methods such as causal inference to analyze observational data as hypothetical RCTs, which have generated similar results to those of randomized trials [13]. Causal inference in observational studies refers to an intellectual discipline which allows researchers to draw causal conclusions based on data by considering the assumptions, study design, and estimation strategies [20]. Causal inference methods, through their well-defined frameworks and assumptions, have the advantage of requiring researchers to be explicit in defining the design intervention, exposure, and confounders, for example through the use of DAGs (Directed Acyclic Graphs) [21], and have helped to overcome concerns about bias in the analysis of observational studies [10]. Moreover, recently, large observational studies have become more popular in the era of big data because of their ability to leverage and analyze multiple sources of observational data [22] such as from population databases, social media, and digital health tools [23]. Another innovation is the E-value, “the minimum strength of association, on the risk ratio scale, that an unmeasured confounder would need to have with both the treatment and the outcome to fully explain away a specific treatment-outcome association, conditional on the measured covariates” [24]. The E-value is an intuitive metric to help determine how robust the results of a study are to unmeasured confounding. A summary of the methods and their application can be seen in Table 2.

**Table 2:** Innovations in randomized controlled trials and observational studies

Randomized controlled trials			
Innovation	Definition	Strengths	Application of the method
Adaptive trials	Adaptive methods include scheduled interim looks at the data during the trial. This leads to predetermined changes based on the analyses of accumulating data, all the while maintaining trial validity and integrity [15]	Despite being more complex than traditional RCTs, adaptive trials can bring about numerous benefits, such as shortening trial duration or obtaining more precise conclusions [15]	Jardine et al. 2022 [25] Wang et al. 2018 [26]
Sequential trials	Sequential trials are an approach to clinical trials during which subjects are serially recruited and study results are continuously analyzed [16]. Once enough data enabling a decision regarding treatment effectiveness is collected, the trial is stopped [17]	Since a sequential trial can be halted as soon as treatment efficacy or lack thereof is demonstrated, a reliable result is obtained with a minimum number of patients [16]	Lewis and Bessen 1990 [16] Gu et al. 2015 [27]
Platform trials	Platform trials are a type of clinical trial during which multiple interventions can be compared simultaneously to a common control group within a single master protocol [28]	With a platform trial, having a common control arm can decrease the number of patients to be enrolled, the cost, and the time of a RCT [29]	Parker et al. 2018 [30] Yee et al. 2022 [31]
EHR (Electronic Health Record)-based clinical trials	EHRs and an expanded access to routinely-collected clinical data has resulted in RCTs being conducted within the context of EHR-based clinical trials. [19]	EHRs may facilitate pre-screening of patients by age, sex, and diagnosis, helping to exclude ineligible patients, and reduce the overall screening duration in clinical trials [32]	Price et al. 2017 [33] Bereznicki et al. 2008 [34]
**Observational studies**			
Innovation	Definition	Strengths	Application of the method
Causal inference methods	Causal inference in observational studies refers to an intellectual discipline which allows researchers to draw causal conclusions based on data by considering the assumptions, study design, and estimation strategies [20]	Causal inference methods, through their well-defined frameworks and assumptions have helped to overcome concerns about bias in the analysis of observational studies [10]	Ekline et al. 2011[35] Skerritt et al. 2021 [36]
DAG (Directed acyclic graph)	When considering the effect of one variable on another, DAGs serve as a visual representation of causal assumptions. This structured approach moves the conversation forward by serving as a visual aid that makes underlying relations explicit [37]	DAGs can help identify possible confounding for the causal question being considered [37]	Pakzad et al. 2023 [38] Byrne et al. 2019 [39]
E-value	The E-value is “the minimum strength of association, on the risk ratio scale, that an unmeasured confounder would need to have with both the treatment and the outcome to fully explain away a specific treatment-outcome association, conditional on the measured covariates” [24]	The E-value is an intuitive metric to help determine how robust the results of a study are to unmeasured confounding [24]	Bender Ignacio et al. 2018 [40] Eastwood et al. 2018 [41]
Use of “big data”	Large observational studies have become more popular in the era of big data because of their ability to leverage and analyze multiple sources of observational data [22] such as from population databases, social media, and digital health tools [23]	Use of big data in research can help with hypothesis generating, and focuses on the temporal stability of the association [23]	Khera et al. 2018 [42] Ahmed et al. 2023 [43]

Despite the salient advances taking place, challenges and future considerations exist for both observational and experimental research methodologies (see Appendix A). One concern is how to apply innovations to new contexts, different topics, and novel areas of research. For example, causal inference methods are widely used in pharmacoepidemiology, but have so far rarely been used in other fields such as primary care [44]. One solution could be to encourage the use of these novel techniques by developing guidelines, sensitizing medical students to these methods by including them in the curriculum, or inclusion of more impartial and open-minded journal review boards. Such measures could facilitate cross-fertilization of methods across disciplines and foster their use in more studies.

## Conclusion

When considering RCTs and observational studies, several key take-home messages can be drawn:

- No study is designed to answer all questions, and consequently, neither RCTs nor observational studies can answer all research questions at all times. Rather, the research question and context should drive the choice of method to be used.
- Both observational studies and RCTs face methodological challenges and are subject to bias. While any single study is flawed, it is the hope that the body of evidence together will show consistency in the effect of the exposure. Furthermore, triangulation of evidence from observational and experimental approaches can furnish a stronger basis for causal inference to better understand the phenomenon studied by the researcher [10].
- Recent methodological innovations in health research represent a paradigm shift in how studies should be planned and conducted [44]. More knowledge translation is needed to disseminate these innovations across the different health research fields.

Finally, RCTs and observational studies can result in evidence that can subsequently improve the health and clinical care for patients, the desired effect and general aim for all researchers, decision-makers, and physicians using these study methods. However, the necessity of RCTs for establishing the highest level of evidence, remains an area of substantial disagreement, and it will be important to continue discussions around these issues going forward.

### Supplementary Information

**Additional file 1. **

## Data Availability

Not applicable.

## Abbreviations

RCT: Randomized controlled trial
AAC: Alan A Cohen
EM: Ellie Murray
EL: Elena Losina
FL: Francois Lamontagne
NS: Nadia Sourial
EHR: Electronic health records
DAG: Directed Acyclic Graph
