# Correction: Rethinking the pros and cons of randomized controlled trials and observational studies in the era of big data and advanced methods: a panel discussion

**DOI:** 10.1186/s12919-024-00299-w

**Published:** 2024-08-16

**Authors:** Pamela Fernainy, Alan A. Cohen, Eleanor Murray, Elena Losina, Francois Lamontagne, Nadia Sourial

**Affiliations:** 1 Department of Health Management, Evaluation and Policy, School of Public Health, University of Montreal, Montreal, QC Canada; 2 Research Centre of the Centre Hospitalier de L’Université de Montréal (CHUM), Montreal, QC Canada; 3 Department of Family and Emergency Medicine, Faculty of Medicine and Health Sciences, University of Sherbrooke, Montreal, QC Canada; 4 CHUS Research Centre, Montreal, QC Canada; 5 Centre de Recherche Sur Le Vieillissement, Montreal, QC Canada; 6 Butler Columbia Aging Center, New York, NY USA; 7 Department of Environmental Health Sciences, Mailman School of Public Health, Columbia University New York, New York, NY USA; 8 School of Public Health, Boston University, Boston, MA USA; 9 Harvard Medical School Department of Orthopedic Surgery, Cambridge, MA USA; 10 Departement de Medicine, University of Sherbrooke, Montreal, QC Canada

**Correction: BMC Proc 18 (Suppl 2), 1 (2024)**

**https://doi.org/10.1186/s12919-023-00285-8**

Following publication of the original article [1], the authors requested to update the funding section. The original and updated funding statements are given below:

Original: This work was funded by a Canadian Institutes of Health Research grant (#178264).

Updated: This work was funded by the Canadian Institutes of Health Research grant (#178264). The authors would like to thank the Quebec Population Health Research Network (QPHRN) for its contribution to the financing of this open-access publication. Ce travail a été financé par une subvention des Instituts de recherche en santé du Canada (#178264). Les auteurs remercient l’axe Politiques publiques et santé des population du Réseau de recherche en santé des populations du Québec (RRSPQ) pour sa contribution au financement de cet article.

The original article [1] has been updated.
