# Supplementary material for: Rethinking the pros and cons of randomized controlled trials and observational studies in the era of big data and advanced methods: a panel discussion
Source: BMC Proc. 2024 Jan 18;18(Suppl 2):1. doi: 10.1186/s12919-023-00285-8 (PMC10795211; doi:10.1186/s12919-023-00285-8)
Supplement: Supplementary file 1 — Additional file 1. [file 12919_2023_285_MOESM1_ESM.pdf]

February 24th 2022

RETHINKING THE PROS AND CONS OF RANDOMIZED TRIALS AND OBSERVATIONAL STUDIES IN THE ERA OF BIG DATA AND ADVANCED METHODS

Panelists

Nadia Sourial, PhD
Alan Cohen, PhD
Ellie Murray, PhD
François Lamontagne, MD
Elena Losina, PhD

RCTs

A randomized controlled trial (RCT) is a study where researchers randomly place participants into an intervention or control group.

Pros	Cons
Randomizing makes the two groups very similar and easy to compare	Can be very costly and take many years to conduct
Helpful to study clinical and pharmacologic interventions	Data collected can be biased due to non-compliance and drop-outs
Current gold standard for studying the effect of an intervention	Easy to overlook biases
Relatively easy to conduct	
Gives the true effect of an intervention under ideal conditions	

Observational studies

An observational study is one where researchers have no control over who receives or does not receive the intervention.

Pros	Cons
Useful to provide real world evidence	Subject to outside factors that could distort the effect of the intervention
Relatively fast and inexpensive to conduct	Can be complex to design
Suitable for studies where randomization is not feasible (e.g. studying policies)	Subject to limitations in the data available
Uses already available data like electronic medical records	Advanced analytical approaches often required

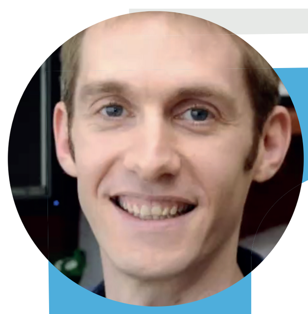

The idea that there is any gold standard design impedes critical thought.

- Alan Cohen

RCTs can provide a false sense of security regarding bias; in observational studies, we are more cognisant of bias and explicit in addressing it.

- Nadia Sourial

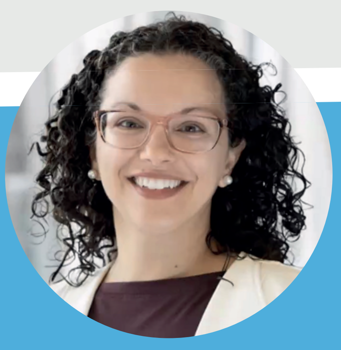

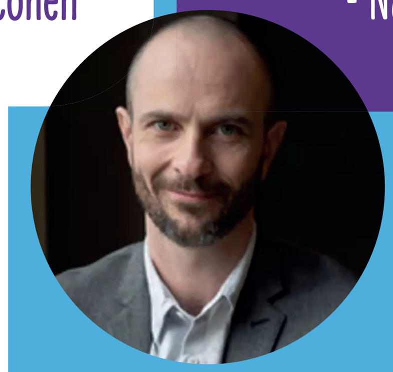

There is no 'free lunch' – RCTs require greater effort during design and planning, while proper analysis of existing observational studies data requires sophisticated analytic expertise.

- Elena Losina

RCTs are really just observational studies with no confounding for randomization & a clearly specified intervention. All the other challenges of observational studies can also happen in RCTs!

- Ellie Murray

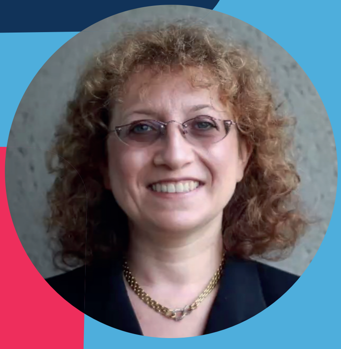

One cannot adjust for what is not measured and what does not vary. If 100% of clinicians administer treatment A, observational studies cannot prove that B would be superior.

- François Lamontagne

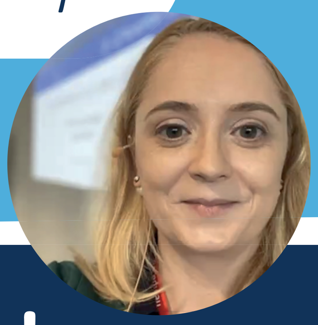

Attendees's quotes

80% of attendees agreed that there are circumstances where observational studies are preferred over RCTs.

"Internal and external validity are not a zero sum game. Both can (and should be) maximized."

"The question is the main driver to the selection of experimental vs. observational designs."

"Biggest advantage of RCTs – equal distribution of both known, and more IMPORTANTLY, unknown factors."

Take home messages

1. Both RCTs and observational studies can fall on a spectrum of good, to bad, to ugly.
2. Both methods should be seen as complementary.
3. Coming to the same conclusion using both methods is an advantage and can lead to an improvement of clinical care.
4. One size never fits all: it's best to think of the questions that one would like to have answered first, and then pick the method that best answers the method.

Special thanks to all participants and our moderator Lise Gauvin!

A webinar sponsored jointly by: CRCHUM Centre de recherche Centre hospitalier de l'Université de Montréal; Centre de recherche sur le vieillissement; Centre de recherche CHUS; UdS Université de Sherbrooke
